# Glucagon-Like Peptide-1 Receptor Agonist Semaglutide Improves Eating Behavior and Glycemic Control in Japanese Obese Type 2 Diabetic Patients

**DOI:** 10.3390/metabo12020147

**Published:** 2022-02-04

**Authors:** Takayuki Masaki, Yoshinori Ozeki, Yuichi Yoshida, Mitsuhiro Okamoto, Shotaro Miyamoto, Koro Gotoh, Hirotaka Shibata

**Affiliations:** Department of Endocrinology, Metabolism, Rheumatology and Nephrology, Faculty of Medicine, Oita University, Yufu City 879-5593, Japan; ozeki23@oita-u.ac.jp (Y.O.); y-yoshida@oita-u.ac.jp (Y.Y.); mokamoto@oita-u.ac.jp (M.O.); shoutarou1029@oita-u.ac.jp (S.M.); gotokoro@oita-u.ac.jp (K.G.); hiro-405@oita-u.ac.jp (H.S.)

**Keywords:** glucagon-like peptide-1, eating behavior, obesity, type 2 diabetes

## Abstract

We evaluated time-course changes and the relationship between eating behavior and glycemic profile during the treatment of 34 obese type 2 diabetic patients with the glucagon-like peptide-1 receptor agonist (GLP1-RA) semaglutide. Changes in dietary habits were evaluated using the Japan Society for the Study of Obesity questionnaire. Semaglutide improved body weight and hemoglobin A1C (HbA1c) 3 and 6 months after treatment. In addition, semaglutide led to marked improvements in the total scores for eating behavior items on the questionnaire. In particular, changes in the scores regarding the sensation of hunger, food preference, eating style, regularity of eating habits and emotional eating behavior were significantly improved during semaglutide treatment. By contrast, there were no significant changes in the scores for the recognition of weight and constitution and external eating behavior. Furthermore, changes in the scores regarding the sensation of hunger and food preference were correlated with changes in HbA1c after semaglutide treatment. Multivariable regression analyses showed that the change in the sensation of hunger was related to HbA1c during treatment. In conclusion, the GLP1-RA semaglutide regulates eating behavior, and, in particular, the sensation of hunger is closely related to the improvement in HbA1c by semaglutide in obese patients with type 2 diabetes.

## 1. Introduction

Obesity and type 2 diabetes mellitus (T2DM) are correlated with several lifestyle factors, including food intake [1,2,3]. Many eating behaviors, such as feeding style, the sensation of hunger, food preference and the regularity of eating habits are associated with the development of T2DM and obesity [4,5]. Changes in eating behavior are important for improving obesity and glycemic metabolism [6,7,8]. Several interventions, including eating behavior, can potentially inform the development of more effective and sustainable interventions for obesity and related metabolic disorders [9,10].

The actions of glucagon-like peptide-1 (GLP-1) in controlling hyperglycemia and appetite are highlighted. GLP-1 controls meal-related hyperglycemia via insulin augmentation and inhibits food intake [11]. GLP-1, a peptide hormone produced in intestinal endocrine L cells [11], is released in response to meal intake, and is rapidly inactivated by dipeptidyl peptidase IV [12]. Because GLP-1 secretion from the gut seems to be impaired in obese patients, GLP-1 secretion may play a role in the pathophysiology of obesity [13]. Along these lines, increased secretion of GLP-1, induced by delivering nutrients to lower parts of the small intestines, may be one factor that explains weight loss and improvements in glycemic control. In addition, GLP-1 affects the cardiovascular system [9,13]. Especially, GLP-1 receptor agonists, such as liraglutide, reduce cardiovascular events in high-risk patients with type 2 diabetes [9,13]. 

GLP-1 receptor (GLP-1R) signaling is a promising target of obesity and type 2 diabetes treatment, which aims to activate intestinal GLP-1- and GLP-1R-expressing central nervous system circuits resulting from normal eating and GLP-1R agonist drug therapy [14]. The GLP-1R agonists can be categorized as either short-acting compounds (e.g., exenatide), which provide short-lived receptor activation, or as long-acting compounds (e.g., dulaglutide), which activate GLP-1R continuously [15]. The pharmacokinetic differences between these drug types lead to important differences in their pharmacodynamic profiles. Short-acting GLP-1R agonists lower postprandial blood glucose levels via the inhibition of gastric emptying, whereas the long-acting compounds have a stronger effect on fasting glucose levels, which is predominantly mediated through their insulinotropic and glucagonostatic actions [15].

The GLP1-R agonist semaglutide regulates food intake, induces weight loss and improves glucose metabolism in obese patients with T2DM [16,17]. Semaglutide has high structural homology to human GLP-1. Native GLP-1 has a short elimination half-life, whereas semaglutide has a long half-life of about 1 week and can be administered once weekly [18]. GLP-1 is a hormone with blood glucose-lowering action during hyperglycemia, and it induces insulin secretion and reduces glucagon secretion in a glucose-dependent manner [19]. The mechanisms underlying its anti-obesity and anti-diabetic actions are partly due to a combination of the effects of GLP-1 on the gastrointestinal tract and the brain [20]. In addition, the anorectic and anti-diabetic effects of GLP-1 are mediated by both the activation of GLP-1R expressed on vagal afferents and GLP-1R activation in the brain [21]. These results indicate that semaglutide influences eating behavior; however, the comprehensive relationship between eating behavior and glycemic profiles during semaglutide treatment is unknown. 

This study evaluated the time-course changes in eating behavior and the relationship between eating behavior and glucose profiles in obese diabetic patients during semaglutide treatment.

## 2. Results

### 2.1. Baseline Characteristics of Participants

Table 1 shows the baseline characteristics of 34 patients. The mean age was 52.8 ± 9.1 years, the BMI was 35.0 ± 6.2 kg/m^2^ and the T2DM duration was 10.1 years. The mean SBP and DBP were 129.9 ± 12.1 mmHg and 76.8 ± 11.5 mmHg, respectively. The mean FPG and HbA1c were 137.5 ± 58.1 mg/dL and 7.3 ± 1.1%, respectively. The mean serum concentrations of LDL, TGs, HDL cholesterol, BUN and Cr were 117.6 ± 30.4 mg/dL, 170.3 ± 84.6 mg/dL, 54.7 ± 15.3 mg/dL, 15.6 ± 7.3 mg/dL and 0.8 ± 0.4 mg/dL, respectively (Table 1). The data for glucose metabolism and obesity were normally distributed, as indicated by the results of the Shapiro–Wilk test (*p* > 0.05 for each).

### 2.2. Time-Course Changes in BW, BMI, and BP after Semaglutide Treatment

Table 1 also shows the time-course changes in BW, BMI and metabolic parameters. A significant reduction in BW was observed after semaglutide treatment (Table 1). The BW was 90.8 ± 16.9 kg before treatment and 86.3 ± 17.9 kg at 6 months after semaglutide treatment (*p* < 0.01). BMI was also reduced at 3 and 6 months after treatment (*p* < 0.01; Table 1). By contrast, the mean SBP and DBP did not significantly change during the study period (*p* > 0.1; Table 1).

### 2.3. Time-Course Changes in Plasma Metabolic Parameters after Semaglutide Treatment

The fasting plasma glucose (FPG) and HbA1c levels were significantly decreased after semaglutide treatment (Table 1). The average FPG levels were 137.5 ± 58.1 mg/dL before treatment and 111.2 ± 28.5 mg/dL at 6 months after treatment (*p* < 0.01; Table 1). The HbA1c levels were 7.3 ± 1.1% before treatment and 6.4 ± 0.9% at 6 months after treatment (*p* < 0.01; Table 1). The fasting TG levels were 170.3 ± 84.6 mg/dL before treatment and 144.8 ± 80.9 mg/dL at 6 months after treatment (*p* < 0.01; Table 1). The fasting LDL levels were 117.6 ± 30.4 mg/dL before treatment and 113.3 ± 28.9 mg/dL at 6 months after treatment (*p* < 0.01; Table 1). By contrast, the HDL levels increased from 54.7 ± 15.3 mg/dL before treatment to 58.1 ± 15.7 mg/dL at 6 months after treatment (*p* < 0.01; Table 1). The blood urea nitrogen (BUN) and Cr levels did not significantly change during the study period (*p* > 0.1; Table 1).

### 2.4. Time-Course Changes in Eating Behavior after Semaglutide Treatment

The effects of semaglutide on eating behavior were evaluated before treatment and 3 and 6 months after treatment. Semaglutide treatment significantly reduced the total score for eating behavior (pre-score: 108.6 ± 18.4, 3 months; 93.6 ± 19.1, 6 months; 92.9 ± 16.7; *p* < 0.01) (Table 2). There were no significant changes in the scores for the recognition of weight and constitution after semaglutide treatment (*p* > 0.1; Table 2). External eating behavior tended to decrease after treatment, but not significantly (pre-score: 20.9 ± 5.2, 3 months; 20.0 ± 5.3, 6 months; 19.8 ± 4.8; *p* > 0.05) (Table 2). Both emotional eating behavior and the sensation of hunger were significantly decreased at 3 and 6 months after treatment (pre-score: 10.3 ± 1.9, 3 months; 6.9 ± 2.3, 6 months; 7.1 ± 2.3; *p* < 0.01) (the sensation of hunger: pre-score: 15.2 ± 3.9, 3 months; 9.5 ± 3.2; 6 months; 10.1 ± 3.3; *p* < 0.01) (Table 2). The scores for eating style, food preference and regularity of eating habits were decreased by semaglutide at 3 and 6 months after treatment (*p* < 0.05 or 0.01) (Table 2). 

### 2.5. Correlation between Changes in Eating Behavior and Changes in BW and Glycemic Metabolic Parameters

Next, we examined the correlations among changes in BW, FBS, HbA1c and eating behavior 3 and 6 months after semaglutide treatment. Changes in the sensation of hunger and food preference were correlated with changes in HbA1c, but not BW and FBS, 3 months after semaglutide treatment (the sensation of hunger: *p* = 0.03, *r* = 0.36; food preference: *p* = 0.03, *r* = −0.36; Table 3). In addition, changes in the sensation of hunger were positively correlated with changes in HbA1c, but not BW and FBS, at 6 months after semaglutide treatment (*p* = 0.04, *r* = 0.34; Table 3). There were no correlations among the changes in LDL, TGs, HDL and eating behavior 3 and 6 months after semaglutide treatment (*p* > 0.1; data not shown).

### 2.6. Multiple Regression Analyses of Changes in BW, FPG, and HbA1c

Multiple regression analyses were conducted to assess the association between HbA1c and eating behavior at 6 months after semaglutide treatment (Table 4). In this study, we chose emotional feeding behavior, the sensation of hunger, eating style, food preference and the regularity of eating habits as candidate eating behaviors by the first screening. Multiple regression analyses were conducted for changes in HbA1c as a dependent variable and changes in eating behaviors as independent variables. The change in the sensation of hunger was an independent determinant of HbA1c in obese diabetic patients after semaglutide treatment in this study (*p* < 0.05; Table 4).

## 3. Discussion

Previous studies have demonstrated the anti-diabetic and anti-obese effects of semaglutide on weight reduction in obese patients with T2DM [16,17]. In the Semaglutide Unabated Sustainability in Treatment of Type 2 Diabetes (SUSTAIN)-1 trial, semaglutide monotherapy significantly reduced glucose metabolism and BW compared to placebo controls [16]. The rates of cardiovascular death, nonfatal myocardial infarction and nonfatal stroke were significantly lower in patients receiving semaglutide than in those receiving the placebo, with improvements in glycemic profiles and weight loss [17]. In agreement with previous studies, this study demonstrated that semaglutide treatment significantly affected both BW and glycemic control in obese patients with T2DM.

Our results also demonstrated the time-course changes in eating behaviors in obese diabetic patients during semaglutide treatment. Compared to the data on eating behavior scores in non-obese healthy subjects in the JASSO database and from other studies [4,5], the scores for eating behavior trended towards those for non-obese healthy subjects, although there are no data regarding healthy control subjects in this study. GLP-1 promotes satiety and suppresses food intake in animals [22] and humans [23,24,25]. Semaglutide not only decreases food intake, but improves eating control, including the intake of high-fat foods [23]. The GLP-1RA liraglutide also reduces the intake of fatty foods [7,8]. Similarly, semaglutide improved the food preference score, including that for the intake of high-fat foods in this study. Dietary fat intake impairs glucose metabolism [26] and is strongly correlated with cardiovascular risk in patients with T2DM [27]. A high intake of saturated fat is associated with T2DM and cardiovascular diseases [28]. Thus, semaglutide may be beneficial in limiting fat intake and cardiovascular outcomes. 

Semaglutide led to improvements in the total scores for eating behavior, which significantly decreased at 3 and 6 months after semaglutide treatment. In particular, semaglutide influenced the sensation of hunger, emotional eating behavior, eating style, food preference and the regularity of eating habits. By contrast, there were no significant changes in the scores for the recognition of weight and constitution and eating style. The JASSO questionnaire included questions on eating habit, specifically the sensation of hunger, emotional eating behavior, eating style, food preference and the regularity of eating habits. The questions that patients answered after semaglutide treatment were regarding the following: the sensation of hunger (“Do you eat a lot until you are full?”), emotional eating behavior (“Do you have a desire to eat when you have nothing to do?”), eating style (“Do you eat fast?”), food preference (“Do you like fatty food?”), and the regularity of eating habits (“Do you eat between meals?”). The results indicated that semaglutide regulated eating behavior and glucose homeostasis in obese patients with T2DM. 

Previous studies have described the effects of the GLP-1 liraglutide on eating behavior in patients with T2DM [4,5]. Treatment with liraglutide improved the scores for eating behavior, food preference, and the urge to eat high-fat foods. Liraglutide did not significantly reduce the score for the sensation of hunger [4]. Another study showed that weight loss significantly correlated with the decrease in scores for the recognition of weight and constitution, the sensation of hunger and eating style [5]. From our observations and previous studies, liraglutide and semaglutide regulate eating behavior in similar, but partly different, ways, possibly due to their different central actions in the brain.

A recent study demonstrated the neural substrates mediating this effect of GLP-1 RAs [29]. Both liraglutide and semaglutide have access to a limited number of brain regions, primarily circumventricular organs and a few sites in the hypothalamus [30]. Interestingly, the access and active sites in the hypothalamus are quite different in liraglutide and semaglutide. Semaglutide more effectively modulates parabrachial (PB) arcuate nucleus signaling in the hypothalamus (ARH), compared to liraglutide. The medial and posterior parts of ARH Agouti-related protein (AgRP) neurons project to the PB [31], and clinical studies have identified that the posterior region of the hypothalamus has a crucial effect on BW [32,33]. This finding could be relevant, considering that semaglutide, but not liraglutide, is present in the posterior ARH, and can further modulate AgRP input to the PB. The differences in access and active sites in the brain between liraglutide and semaglutide may influence eating behavior.

The relationships between eating habits and glycemic profiles by GLP-1RA treatment have not been examined to date. In this study, we examined the relationship between changes in eating behavior and FPG and HbA1c in Japanese obese diabetic patients for 3 and 6 months following semaglutide treatment. Changes in the sensation of hunger and food preference were correlated with changes in HbA1c up to 3 and 6 months after semaglutide treatment. Multivariable regression analyses showed that changes in the sensation of hunger were independent and significant determinants of changes in HbA1c.

This finding indicates that the sensation of hunger is a key factor for improving HbA1c during semaglutide treatment in obese patients with T2DM. The sensation of hunger is correlated with several molecular mechanisms in the brain [2,3]. Eating behavior, such as the recognition of weight and constitution, is involved in the cerebral cortex and limbic system, rather than the hypothalamus and brainstem. By contrast, the sensation of hunger is controlled by the hypothalamus and brainstem. Several studies have shown that GLP-1 effectively acts on the hypothalamus and brainstem [29]. It is possible that the sensation of hunger is correlated with hypothalamus and brainstem activation during GLP-1RA treatment. 

Interestingly, this study found no correlations among changes in FBS, BW and eating behavior 3 and 6 months after semaglutide treatment. This may be due to the short-term nature of the study. In addition, weight loss may be regulated by an increase in energy expenditure, rather than by eating behavior. A large, long-term study is needed to examine the relationships among BW, FBS and eating behavior.

This study had some limitations. Firstly, the study was hampered by the small sample size and lack of hard data. Many variables/parameters were measured in a few subjects, and the statistical post hoc power calculation was low. Secondly, the study design did not allow us to examine a causal relationship. It is possible that weight loss alone influenced eating behavior, rather than the effects of semaglutide. Thirdly, although we did not change the medical treatment in this study, drug interactions between semaglutide and other medications might have influenced the outcome. In fact, the eating behavior score tended to increase upon treatment with semaglutide and a sodium-glucose transport protein 2 inhibitor, compared to semaglutide alone (T.M., unpublished data). Future large, prospective studies, which include appropriate control patients, would help to clarify some of the outcomes. The strength of this study is that the objective methods used to estimate the changes in eating behavior recorded by the subjective questionnaire method were meaningful. 

In summary, this is the first study to demonstrate the relationships among the GLP1-RA semaglutide, glucose metabolism and eating behavior. We conclude that the GLP1-RA semaglutide regulates eating behavior, and, in particular, the sensation of hunger is related to an improvement in HbA1c by semaglutide in obese patients with T2DM.

## 4. Materials and Methods

### 4.1. Patients

We retrospectively recruited 34 obese diabetic patients (12 males and 22 females; mean body mass index (BMI), 35.0 ± 6.2 kg/m^2^; age, 52.8 ± 9.1 years) undergoing semaglutide treatment at Oita University Hospital (Yufu, Japan) from August 2020 to August 2021. Patients were selected for semaglutide treatment according to the standard drug information in Japan. In total, 19 of the 34 patients with hypertension were taking an antihypertensive drug (calcium channel inhibitor, 8 patients; angiotensin receptor blocker, 9 patients; others, 2 patients); 9 of the 34 patients with hyperlipidemia were taking lipid-lowering medication (statins, 7 patients; fibrates, 1 patient; others, 1 patient); all 34 patients were taking glucose-lowering medication (insulin, 0 patients; metformin, 12 patients; dipeptidyl peptidase 4 inhibitor, 8 patients; sodium-glucose transport protein 2 inhibitor, 6 patients; sulfonylureas, 2 patients; others, 7 patients). Six patients had diabetic neuropathy, five had diabetic retinopathy, three had diabetic nephropathy and two patients had chronic cerebral infarction. There were no significant changes in diabetes complications during the study period. None of the patients had organic heart disease as determined by physical examination, chest X-ray, electrocardiography and echocardiography, nor did they have a history of myocardial infarction. Extensive clinical and hormonal endocrine evaluations were used to identify and exclude patients with endocrine diseases.

During this study, we did not change the medical treatments outlined above. According to standard drug information, semaglutide was started at 0.25 mg/week, which was increased by 0.25 mg/week to a final dose of 0.5 mg/week, the standard dose in Japan. Treatment with semaglutide was decided by each attending physician. The patients with T2DM were treated for the first time with semaglutide between August 2020 and August 2021. Extensive clinical and hormonal endocrine evaluations were used to identify and exclude patients with endocrine diseases and active cardiovascular diseases. The study was conducted in accordance with the Declaration of Helsinki and was approved by the Ethics Committee of Oita University (identification code 762). The patients provided informed consent to participate in the study.

### 4.2. Anthropometry and Body Analyses

Body weight (BW), height and BMI were measured in all patients. BW was measured to the nearest 0.1 kg using digital scales, height was measured to the nearest 0.1 cm with the patients wearing light indoor clothing, and BMI was calculated using the patient’s weight and height (kg/m^2^).

### 4.3. Blood Sampling and Analysis of Blood Pressure

Blood was drawn from 8:00 to 11:00 a.m. from the antecubital vein of the patients in a recumbent position after an overnight fast. The patients underwent routine laboratory tests, including assays for plasma levels of low-density lipoprotein (LDL), triglycerides (TGs), high-density lipoprotein (HDL), blood urea nitrogen (BUN) and creatinine (Cr). Fasting plasma glucose (FPG) and hemoglobin A1c (HbA1c) were measured via high-performance liquid chromatography. Systolic blood pressure (SBP) and diastolic blood pressure (DBP) were measured using the cuff oscillometric method between 8:00 and 11:00 a.m. Anthropometry, blood sampling and analysis of BP were performed in accordance with previous studies [34,35]. 

### 4.4. Assessment of Eating Behavior

Eating behavior was quantified using the Japan Society for the Study of Obesity (JASSO) questionnaire [36] before and after semaglutide treatment. The seven eating behavior items are as follows: (1) recognition of weight and constitution; (2) external eating behavior (e.g., “When you walk past the supermarket, do you have the desire to buy something delicious?” and “If you see others eating, do you also have the desire to eat?”); (3) emotional eating behavior (e.g., “Do you have the desire to eat when you are irritated?” and “Do you have a desire to eat when you have nothing to do?”); (4) sensation of hunger (e.g., “Do you get irritated when you feel hungry?” and “Do you often have regret because you have eaten a lot of food?”); (5) eating style (e.g., “Do you eat fast?” and “Are you known to eat a lot of food?”); (6) food preference (e.g., “Do you often snack on bread?,” “Do you like meat?” and “Do you like noodles?”); (7) regularity of eating habits (e.g., “Is your dinner time too late at night?” and “Do you gain body weight during the holidays?”). All items were rated on a four-point scale ranging from 1 (seldom) to 4 (very often).

### 4.5. Statistical Analyses

The data are presented as the mean ± standard deviation, and were analyzed using commercial software (JMP Pro 14.1; SAS Institute, Cary, NC, USA). The time-course change for each parameter was statistically evaluated using the one-way ANOVA test. We examined the data to confirm the normality of the data distribution using the Shapiro–Wilk test prior to the ANOVA test (JMP Pro 14.1; SAS Institute, Cary, NC, USA). *p* < 0.05 was considered statistically significant. Next, we calculated the correlation coefficients among changes in BW, FBS, HbA1c and eating behavior 3 and 6 months after semaglutide treatment by simple regression analyses. Finally, multiple regression analyses were conducted for changes in HbA1c as a dependent variable, and changes in eating behaviors as independent variables.

## 5. Conclusions

In conclusion, the GLP1-RA semaglutide regulates eating behavior, and eating behavior is related to improvements in HbA1c in obese patients with T2DM by GLP1-RA semaglutide.

## Figures and Tables

**Table 1:** Basal and time-course changes in body weight and plasma metabolic parameters.

	Baseline	3 Month	6 Month	*p*
Age (years)	52.8 ± 9.1			
Male/Female	12/22			
Body weight (kg)	90.8 ± 16.9	87.8 ± 17.0	86.3 ± 17.9	<0.01
Total body weight loss (kg)		3.0 ± 1.9	4.5 ± 1.2	
BMI (kg/m^2^)	35.0. ± 6.2	33.8 ± 6.1	33.2 ± 6.1	<0.01
Systolic blood pressure (mmHg)	129.9 ± 12.1	126.7 ± 11.9	127.8 ± 11.3	N.S
Diastolic blood pressure (mmHg)	76.8 ± 11.5	77.2 ± 12.6	75.4 ± 10.0	N.S
Fasting plasma glucose (mg/dL)	137.5 ± 58.1	110.7 ± 26.7	111.2 ± 28.5	<0.01
HbA1c (%)	7.3 ± 1.1	6.7 ± 1.0	6.4 ± 0.9	<0.01
Triglycerides (mg/dL)	170.3 ± 84.6	154.6 ± 77.5	144.8 ± 80.9	<0.01
HDL cholesterol (mg/dL)	54.7 ± 15.3	55.8 ± 15.5	58.1 ± 15.7	<0.01
LDL cholesterol (mg/dL)	117.6 ± 30.4	108.3 ± 28.0	113.3 ± 28.9	<0.01
BUN (mg/dL)	15.6 ± 7.3	15.6 ± 8.4	15.9 ± 6.6	N.S
Creatinine (mg/dL)	0.8 ± 0.4	0.8 ± 0.4	0.8 ± 0.3	N.S

The data are presented as the mean ± standard deviation and assessed by one-way ANOVA test. N.S: not significant.

**Table 2:** Basal and time-course changes in eating behavior.

	Baseline	3 Month	6 Month	*p*
Recognition of weight	16.5 ± 3.3	16.2 ± 3.5	15.2 ± 3.4	N.S
External eating behavior	20.9 ± 5.2	20.0 ± 5.3	19.8 ± 4.8	N.S
Emotional eating behavior	10.3 ± 1.9	6.9 ± 2.3	7.1 ± 2.3	<0.01
Sense of hunger	15.2 ± 3.9	9.5 ± 3.2	10.1 ± 3.3	<0.01
Eating style	11.0. ± 3.7	9.5 ± 3.1	9.2 ± 3.1	<0.01
Food preference	17.3 ± 5.4	16.2 ± 5.4	15.5 ± 4.8	<0.05
Regularity of eating habits	17.2 ± 3.6	15.1 ± 3.3	15.4 ± 3.3	<0.01
Total score	108.6 ± 18.4	93.6 ± 19.1	92.9 ± 16.7	<0.01

The data are presented as the mean ± standard deviation and assessed by one-way ANOVA test. N.S: not significant.

**Table 3:** Correlation between eating behavior and delta-BW, FPG and HbA1c at 3 and 6 months.

	BW		FPG		HbA1c	
Variables (Delta)	*r*	*p* Value	*r*	*p* Value	*r*	*p* Value
Variables (0–3 month)						
Recognition of weight	0.14	0.41	−0.02	0.89	−0.17	0.33
External eating behavior	0.29	0.09	0.07	0.42	−0.09	0.60
Emotional eating behavior	0.01	0.93	0.01	0.91	−0.05	0.76
Sense of hunger	−0.05	0.76	0.17	0.32	0.36	0.03 *
Eating style	0.20	0.25	0.11	0.54	0.18	0.29
Food preference	0.01	0.93	0.01	0.93	−0.36	0.03 *
Regularity of eating habits	0.13	0.46	−0.17	0.33	−0.02	0.88
Variables (0–6 month)						
Recognition of weight	0.04	0.79	−0.03	0.83	−0.20	0.24
External eating behavior	0.21	0.21	0.02	0.88	0.09	0.58
Emotional eating behavior	0.10	0.54	0.10	0.54	0.05	0.80
Sense of hunger	0.10	0.54	0.25	0.14	0.34	0.04 *
Eating style	0.25	0.14	0.04	0.78	0.13	0.46
Food preference	0.17	0.31	0.04	0.79	−0.07	0.71
Regularity of eating habits	0.08	0.63	−0.13	0.45	−0.14	0.41

Variables: variables at 3- and 6-month treatment: body weight (BW), fasting plasma glucose (FPG) and hemoglobin A1c (HbA1c). *r* correlation coefficient, * *p* < 0.05 significant correlation between factors.

**Table 4:** Multiple linear regression models with delta-HbA1c as the dependent variable.

Variables (Delta)	*r*	*t* Value	*p* Value
Emotional eating behavior	0.07	0.33	0.74
Sense of hunger	0.38	2.13	0.04 *
Food preference	0.05	0.21	0.83
Eating style	−0.29	−1.31	0.67
Regularity of eating habits	0.08	0.42	0.20

*r* correlation coefficient, * *p* < 0.05 significant correlation between factors.

## Data Availability

The data presented in this study are available on reasonable request from the corresponding author. The data are not publicly available due to preserve patient confidentiality and privacy.
